# The Doctorate Address, Delivered at the 40th Annual Commencement Exercises of Rush Medical College

**Published:** 1883-04

**Authors:** Moses Gunn

**Affiliations:** Professor of Surgery and Clinical Surgery, Rush Medical College


CHICAGO MEDICAL

### JOURNAL & EXAMINER.

VOL. XLVI.—APRIL, 1883.—No. 4.

### Original Communications.

### ARTICLE I.

THE DOCTORATE ADDRESS, Delivered at the Fortieth Annual Commencement Exercises of Rush Medical College, held in Central Music Hall, Chicago, February 20, 1883, by MOSES GUNN, M.D., LL.D., Professor of Surgery and Clinical Surgery, Rush Medical College.

GENTLEMEN OF THE GRADUATING CLASS:

As in nature, full fruition culminates a series of developmental changes which originated in the germ, so in education, the ceremonies of graduation typify that grade of development which enables the graduate to commence the enjoyment of the fruit of knowledge which he has thus far acquired. And as in nature the fruit contains the germ of future representatives of the family, which are to be an improvement upon or a degeneration from their predecessors, according to the management of the germ in its developmental progress, so in education, the acquired knowledge of the graduate contains the germ which may perpetuate and increase human knowledge, or degenerate and die, according to the treatment which it shall receive.

You, gentlemen, have now gathered your first harvest from the medical tree, and while the fruit of that harvest may justly contribute to your delectation and profit inyour future career, remember that it contains a rich supply of germs, some of which it behooves you to cultivate and improve.

It is the duty of every man to do good in his day and generation according to his ability; to leave his race, at least, unimpoverished by his existence. It is a privilege which may be enjoyed by every man of average health and intellect, to finally leave the world some better for his having lived in it. This general proposition applies to the grand body of human knowledge, as well as to the material wealth of the world.

The faithful propagation of a few or many of the germs contained in the fruit which you have thus far plucked will enable you to discharge the duty and enjoy the privilege which I have indicated. To do the first implies work, much work. Idleness will leave the balance in your account with the world against you, and rest assured that the world will torment you with frequent reminders of your indebtedness, though it fails to collect the debt.

To accomplish the second, you should be constant in your devotion to the science which you have chosen, using it as a means of doing good to the world at large, and to your profession in particular.

The germs which you find in your fruit must be carefully guarded against the mildew of forgetfulness on the one hand, and the scorching effects of sensual pursuits on the other, till you are in a situation to plant and cultivate them; and when that seed and cultivation time shall come—and it should begin even now—remember that your harvest will not be rich unless your husbandry shall have been thorough.

But though you neglect and waste the germs, you may not even selfishly enjoy your fruit without bestowing upon it an assiduous and protecting care. The fruit of the tree of knowledge which the student gathers is extremely perishable, unless it receives constant protection. Studious habits and thoughtful application only will keep off the spores floating thickly in the atmosphere of idleness, which, settling down upon your store, develop the teeming bacterian myriads of decay and putrescence.

It grieves me somewhat that in falling into this figurative expression, in accordance with prevalent and fashionable modes of thought, I seemingly ignore the pure chemistry of decay ; but I beg you to acquit me of any such dereliction, and to remember, as a standing caution, that all that is new is not always true, and that all that is true is not necessarily new.

But whatever simile may be employed, the marked and stern fact you must appreciate, that your stock of acquired knowledge will rapidly melt away without that reinforcement which comes of studious habits.

But it is not my intention to mar your enjoyment of this era in your lives by reiterating injunctions to labor. An occasion like this should, perhaps, be regarded as a breathing-time in the continuous work of professional life ; a time to forget for a few days that you are laborers under an unrelenting taskmaster.

Let us turn, then, to the consideration of another subject of hardly less importance to the members of a learned profession. *Noblesse oblige.* Your rank and position impose upon you obligations which may not be avoided. The amenities of professional life and intercourse must be faithfully observed, and to observe them promptly and constantly you must be familiar with the principles upon which they are founded. Nay, more; those principles must become a part of your nature. To a certain extent, and in a limited number of men, they are innate. The man who is by nature a gentleman has an instinctive appreciation of them, and such a man will not often greatly err in his dealings with his professional brethren, individually or collectively. But the average man, however humiliating the confession may be, is not naturally a gentleman ; and we cannot shut our eyes to the fact that many average men study medicine, practice medicine, and are widely useful to the public and the profession. Nature's noblemen are not very numerous, and those made by royal letters-patent only, are necessarily extremely defective. Royal letters are sometimes false labels. Men cannot be decreed nor legislated gentlemen. Education only can supplement natural deficiencies in this, as in all other attributes. Hence, ethics justly demands your thoughtful attention, and should be thoroughly studied and consciously practiced, provided always, that it is practiced ethically. Perhaps the most unethical of all men is, generally speaking, he who is always ostentatiously ethical. His crime (for it is a crime) is that of scrupulously observing the letter, while violating the spirit of the law.

Principles of justice, tempered by kindness and graced by benevolence and beneficence, have been invoked for the regulation of professional conduct and intercourse, and thus has grown a body of law known and recognized as the code of medical ethics. This code may, like the body of common law, be unwritten, *i. e.*, it may have never been formally enacted by any law-making power, but its mandates are even more potent than a statute, for the statute may not venture to contravene the principles of common law. Like the English constitution, which is the bulwark of the sturdy Briton, it may never have been formally enacted by parliament, legislature or convention; yet, as the principles of the English constitution could gain no potency by such a process, so the principles of the code need not the force of an enactment. Thus, the medical profession in England (and, I believe, in every other European country) has no written code of ethics; and yet the principles which govern professional intercourse and deportment are as well known and as thoroughly potent as though the talismanic "Be it enacted by the British Medical Association" had attempted its supererogatory work.

Perhaps the earliest approach to a written code of medical ethics may be found in the Hippocratic oath. This oath was imposed by the physician upon his pupil at the time of his indenture, and made the pupil a sworn member of the corporation. It is as follows:

"I swear by Apollo the physician, and Æsculapius, and Health, and All-heal, and all the gods and goddesses, that, according to my ability and judgment, I will keep this oath and this stipulation—to reckon him who taught me this art equally dear to me as my parents; to share my substance with him; to relieve his necessities if required; to look upon his offspring in the same footing as my own brothers, and to teach them this art, if they shall wish to learn it, without fee or stipulation; and that by precept, lecture, and every other mode of instruction, I will impart a knowledge of the art to my own sons, and those of my teachers, and to disciples bound by a stipulation and oath according to the law of medicine, but to none others. I will follow that system of regimen which, according to my ability and judgment, I consider for the benefit of my patients, and abstain from whatever is deleterious and mischievous. I will give no deadly medicine to any one if asked, nor suggest any such counsel; and in like manner I will not give to a woman a pessary to produce abortion. With purity and with holiness I will pass my life and practice my art. I will not cut persons laboring under stone, but will leave this to be done by men who are practitioners of this work. Into whatever houses I enter, I will go into them for the benefit of the sick, and will abstain from every voluntary act of mischief and corruption; and further, from the seduction of females or males, of freemen and slaves. Whatever, in connection with my professional practice, or not in connection with it, I see or hear in the life of men, which ought not to be spoken of abroad, I will not divulge, as reckoning that all such should be kept secret. While I continue to keep this oath unviolated, may it be granted me to enjoy life and the practice of the art, respected by all men, in all times! But should I trespass and violate this oath, may the reverse be my lot!"

It will be noticed, that while thus closely bound to one another, and to practice their art for the good of mankind and the protection of society, guided, in the main, by unselfish and generous motives, still, some of the requirements would hardly pass muster at the present time. It was a very close corporation, which sought to keep the art within certain families, hardly in harmony with the generous method and practice of making doctors in this year of grace which ushers you into the medical profession. Evidently, too, the departments of surgery and gynæcology had not yet been recognized. Methinks those old masters, if their slumber is not too profound, must groan in spirit, if not in body, and their old crumbled bones metaphorically quake at the high-handed procedures of the last half of the nineteenth century. Even the pure and unsanguinary physician of the present day must occasionally shock that holy dust, as he deals out the potentials of our materia medica.

Times have certainly changed! and times will continue to change. The principles of ethics, like the grand principle of the golden rule, will endure, but changed times may effect wonderful change in their application. Perhaps, centuries hence, nay, decades hence, some of the regulations of our code will seem as strange to the medical mind as do now some of the restrictions of the Hippocratic oath.

Our Federal Union of States has a written constitution, formally enacted. The individual States have each its formally adopted constitution. The medical profession in the United States has a code of ethics, formally enacted by the American Medical Association, which regulates professional legislation and conduct as potently as does the constitution of the United States the legislation of Congress. The State societies generally, and even local societies often, formally re enact and adopt this code for their own guidance. It therefore behooves the medical man to familiarize himself with this instrument. This is a duty which is as imperative as that which naturally rests upon every intelligent citizen to understand the principles of the Federal constitution.

The details of the code are arranged in three principal groups:

*First.* The duties of physicians to their patients, and the obligations of patients to their physicians.

*Second.* The duties of physicians to each other, and the profession at large.

*Third.* The duties of the profession to the public, and the obligations of the public to the profession.

Let me invite your attention to some of the subjects considered in this arrangement of articles and sections.

The first group consists of two articles.

Article I is composed of seven sections, treating of the duties of physicians to their patients, and is a comprehensive and sensible exposition, which you will do well to study and practice.

Article II consists of ten sections, devoted to a setting forth of the duties which patients owe to their physicians.

A code regulating the conduct of the members of a corporation may be very properly enacted by that body ; but when its enactments contemplate the regulation of the conduct of other bodies, or of the general public, or of individuals outside of the corporation, it sounds very like a bull directed to that most erratic of all bodies, a comet. It may contain much wisdom and justice, but it is wholly inoperative, and falls little short of the ridiculous. Can it be that, through a process of unconscious cerebration, the framers of the code were emulating Don Quixote in their endeavor to regulate that which is notoriously not to be regulated? Contemplate the American Medical Association enacting that "Even the female sex should never allow feelings of shame or delicacy to prevent their disclosing the seat, symptoms and causes of complaints peculiar to them;" that "a patient should never weary his physician with a tedious detail of events or matters not appertaining to his disease;" that "patients should always, when practicable, send for their physician in the morning, before his usual hour of going out;" that "patients should also avoid calling on their medical adviser unnecessarily during the hours devoted to meals or sleep;" that "a patient should, after his recovery, entertain a just and enduring sense of the services rendered him by his physician!" This is all very well and very true; but as well might they have enacted that all men ought to be sensible, considerate and just.

The conduct of patients relative to their maladies and medical attendant will be just what the joint peculiarities of physician and patient make it. It is a matter over which codes are utterly impotent. Imagine a doctor reading the riot act to the patient who persists in sending for him at unseemly hours! or to one who, having secured his services, fails properly to appreciate them! or to a shrinking, delicate female, who can't tell all the truth at once! Seriously, this article of the code is an excrescence, unsightly and ridiculous, which requires a surgeon's service, extirpation.

The second group, which relates exclusively to members of the profession, individually and collectively, consists of seven articles, containing twenty-nine sections which cover pretty completely the whole ground, and if you follow the letter and spirit of this portion of the code, you can never be far out of the way. I say letter and spirit, for the designing and unscrupulous man may, in many ways, murder the spirit, while observing the letter of the code. Constitutions, and laws with their penalties, will not make all men good, nor make any man all good. The man is good or bad by virtue of his own attributes; not by virtue of laws and enactments. The good man uses the law for his guidance in cases of doubt; the bad one uses it to ascertain just how far he can wander within its limits, or to determine whether life has for him more attractions within or without the pale of the law. Doctors are no exception to the general rule. You will encounter many who know little of the code, but who, apparently, carry within their own consciousness the spirit and actuating force of all law. You will also come in contact with those who are familiar with all its articles and sections, whom you will learn not to trust.

The chief interest, at the present time, is found in Article IV of this group, which pertains to consultations, and that interest centers in the first section, which reads as follows:

"A regular medical education furnishes the only presumptive evidence of professional abilities and acquirements, and ought to be the only acknowledged right of an individual to the exercise and honors of his profession. Nevertheless, as in consultations the good of the patient is the sole object in view, and this is often dependent on personal confidence, no intelligent regular practitioner, who has a license to practice from some medical board of known and acknowledged respectability, recognized by this Association, and who is in good moral and professional standing in the place where he resides, should be fastidiously excluded from fellowship, or his aid refused in consultation, when it is requested by the patient. But no one can be considered as a regular practitioner or fit associate in consultation, whose practice is based on an exclusive dogma, to the rejection of the accumulated experience of the profession, and the aids actually furnished by anatomy, physiology, pathology and organic chemistry."

Early in the summer of 1881 Lord Beaconsfield died, and during his last sickness, the question of a consultation between his irregular medical attendant and certain members of the regular profession became one of general interest to the English profession.

Just what the facts were, I may be excused from knowing, when, in reply to a letter of inquiry, one of the most prominent surgeons of London writes: "I cannot exactly say what were the facts, the really facts, in the case of Lord Beaconsfield, for many things supposed to be facts were only statements made in general conversation, and were probably not more nearly correct than such statements usually are." From this same letter, and from the English journals of that year, we know that the English profession became deeply interested in the question whether a regular physician could, with propriety, meet in consultation an irregular who stated that he had frequently treated his patients in accordance with the principles of regular medicine, and would thus continue to treat Lord Beaconsfield. The affairs of the International Medical Congress, however, submerged the question for a time, but at the meeting of the British Medical Association which followed immediately upon the adjournment of the congress, the presidential address, the address of the president of the medical section, and that of the president of the surgical section, all took up the question. Mr. Barrow, in the presidential address, took the ground that when the irregular practitioner had "consented to place himself under the direction of the consultant, no blame could be attached to the latter in responding to the invitation to take charge of the case."

Dr. Bristow, in his address on medicine, and Mr. Hutchinson, in his address on surgery, both favored consultations with certain irregulars.

Such opinions, from such sources, aroused correspondents and editors to active and earnest discussion, the general sentiment being against the position assumed by the authors of the three addresses. But the Lancashire and Cheshire branch of the association refused to affirm the "*lex non scripta*" of the profession covering the point in question.

The Royal College of Physicians in due time passed upon the subject in the following explicit and dignified language: "While the college has no desire to fetter the opinion of its members in reference to any theory they may see fit to adopt in connection with the practice of medicine, it nevertheless considers it desirable to express its opinion that the assumption or acceptation by members of the profession, of designations implying the adoption of special modes of treatment, is opposed to those principles of freedom and dignity of the profession which should govern the relations of its members to each other and the public. The College, therefore, expects that all its fellows, members and licentiates will uphold those principles by discountenancing those who trade upon such designations."

In the meantime, professional thought in our own country was engaged in a consideration of the superfluously extended code of the American Medical Association. The State Society of New York had, at its annual meeting in February, 1881, appointed a committee to revise the code. The report of that committee was adopted at the annual meeting in February, 1882, and consisted in the presentation of what was practically a substitute for the code. The new code thus adopted wisely omits both the articles which attempt to regulate and determine the obligations of patients and the public to the profession, and correctly confines itself to its proper business, viz., the regulation of professional intercourse.

The chief feature of interest here, as in the code of the American Medical Association, is found in that section which specifies whom the members of the society may meet in consultation. It is as follows:

"Members of the Medical Society of the State of New York and of medical societies in affiliation therewith, may meet in consultation legally qualified practitioners of medicine. Emergencies may occur in which all restrictions should, in the judgment of the practitioner, yield to the demands of humanity."

Thus, that which was only proposed by the three distinguished men in Great Britain, is boldly adopted as a rule of action by the State Society of New York. The Empire State Society thus arrayed itself against the American Medical Association, and, as a natural consequence, its delegates were refused admission to that body at its annual meeting, held at St. Paul in June last.

Three weeks since, the Medical Society of the county of New York met for the purpose of securing an expression of opinion by its members relative to the new code of the State Society, and a resolution approving of the same was adopted by a more than two-thirds vote, viz., 147 to 60.

Since then the regular annual meeting of the State Society has taken place, during which the Society confirmed its action of the previous year.

This bit of history is instructive. It plainly shows that time and altered circumstances are continually changing the relations of men to one another, even in professional aspects.

The altered circumstances consist in the practical and often openly avowed abandonment of their dogmas on the part of the irregulars and an effort, conspicuously ludicrous it is true, to supplement their irregularities by a feeble show of science in their educational methods.

The State, too, lends its potent aid in changing relations. Uneasy gentlemen in the regular ranks clamor for a law which shall purge community of quackery of all forms and elevate the status of the profession by legal enactment. Thus, State boards are created, with sole power to license practitioners of medicine. In the accomplishment of this magnificent feat is illustrated in a forcible manner the truth of the old maxim that "politics make strange bed-fellows." The virtuous and enthusiastic regular, in his desire to purify community of quackery joins cordial hands with the most seductive of all forms of quackery, in order to secure a State licensing board composed of regulars and representatives of such forms of quackery as have numerical strength enough to make their aid desirable in this grand work of reformation. The old Æsculapian lion, graciously and benignantly inclining his august head, and the innocent (?) lambs of empty dogmatism, suspending for the nonce their amusing gambols, lie down together and are led by the monstrous child of folly and error.

Thus, the invocation of the State power accomplishes the legal parity and intimate association of the regular and irregular. Truly, a magnificent and pride-inspiring result!

Is it strange, then, that under these circumstances, in reference to the consultation question, men are coming forward with the proposition to throw down all restrictions and leave the individual practitioner to follow the dictates of his own judgment?

Contemplate, for a single moment, the present status of the medical profession in Illinois. Any member of the profession who has not the good fortune to have been in practice in the State for ten years previous to the first of July, 1877, is obliged to obtain a license to practice from the State Licensing Board, which board is composed alike of regulars and irregulars, doctors of medicine and laymen.

Now, as to the ethics of this arrangement. Dr. A, a graduate of a respectable medical college, presents his diploma and asks for a license to practice his profession. The authority and reputation of the college are pronounced good, and he receives a document authorizing him to go forth with healing on his wings, signed, among others, by Drs. B. and C. irregulars. His first patient, perhaps, desires the counsel of Dr. B. or C. with whom, by virtue of the code, Dr. A. cannot consult, although his authority to practice is derived in part from these very men who have sat in solemn judgment over his own qualifications! Who would not be a member of a legally purified profession? Who would not court the happy lot of one who practices by grace of Drs. B. and C. and who is restrained by the code from meeting them upon a platform to which they have assisted in legally raising him? O, wise law! O, wise code! O, wise members of a learned profession who had not confidence enough in that profession to permit it to stand upon its own merits, but invoked the power of the State to accomplish this most ridiculous and humiliating result! Who would not be a member of the medical profession in Illinois, when to practice his calling he must have the endorsement of a mongrel body, or in event of his ten years' practice previous to 1877, enjoy his independence of that potential body in common with numerous "Lock Hospital" and "Lost manhood" quacks?

You, gentlemen, who contemplate practice in this State, will be compelled to recognize irregular medicine, inasmuch as you cannot legally write the first prescription, nor dress the first wound, without soliciting authority to do so from a body composed in part of avowed irregular practitioners. Old and eminent practitioners throughout the State, and several of your professors in Rush College, are independent of that body only by the operation of a proviso which legally places by their side some of the most notorious and ignorant quacks in the State. Thus the State law *purifies* the profession by legalizing the most shameful quackery. Our only consolation is in the fact that no voluntary act of our own determines our legal status. This comfort, unfortunately, is denied you, for you are compelled to solicit and accept permission to practice from a mongrel board, and thus, by such enforced volition (if I may indulge in the paradox) you are obliged to recognize the legal professional status of men whom the code prohibits you from meeting at the bedside.

But the complications do not end here. The honorable gentlemen of the State Board who represent the regular profession are in frequent consultation with the irregular element of that board, over the qualifications of applicants for license; and I submit the question whether, in so doing, they are not violating the code as essentially as if they were consulting over the treatment of a patient? Nor is this all. The licensing power of the board is an *ex officio* power held by the State Board of Health, a body which meets, consults and acts in behalf of the general public health; which has communities for patients, and over these illustrious patients meets in formal and prolonged consultation; and I again submit the question of violation of the code; is not the degree of violation proportionate to the interests at stake?

If the regular members of the board were to meet in consultation at the bedside of an afflicted patient with the irregular members, they would undoubtedly violate the mandate of the code covering this subject; and when they meet in consultation over afflicted and pest-ridden communities, the principle remains unchanged; and if the National Association refuses admission to a delegate from the New York State Society, can it consistently admit a representative from the Illinois State Board of Health? The delegate from the New York State Society may have never personally violated the code, while the representative of the Illinois State Board of Health is a constant violator.

But I have not reached the climax of this ludicrous and colossal inconsistency. The National Board of Health has also its irregular element, and the distinguished and honorable gentlemen who represent regular medicine in that board are, consequently, in open violation of the code. It cannot be urged that there is no question of therapeutics involved; that the subject of public hygiene offers no opportunity for clashing of fundamental principles. This question of therapeutics was waived in Lord Beaconsfield's case; but the Royal College decided that the offence lay in the professional recognition of men who trade on their qualifying designation; and the representatives of regular medicine in the National and Illinois Boards of Health are in constant professional recognition of men who thus trade on their designation.

It may, perhaps, be said that the association of these men on the board is not professional; that they are merely lay members, as is the case when other professions are represented. But this will not do; for in every instance, the irregular has been appointed to his position because he was an irregular, and represented his peculiar class. Had he not been an irregular he would never have received the appointment, but would have been set aside for some one who could represent the class to which he belonged.

Having now indicated the position which the representatives of regular medicine occupy on these boards, I desire to disclaim any intention or disposition, even, to arraign them for any violation whatever. On the contrary, I repose the fullest confidence in their professional honor and uprightness. I have no fear that they will become contaminated, nor that the medical profession will suffer in any way from their relations to their irrrgular associates.

But I do arraign the medical profession for gross inconsistency. The professional sentiment which overlooks the position of these representatives of regular medicine in the various boards of health where irregular medicine is also represented, and disciplines a society which adopts the rule that its members may consult with "legally qualified practitioners of medicine" and that "emergencies may occur in which all restrictions should, in the judgment of the practitioner yield to the demands of humanity," is inconsistent.

State power has intervened in each instance, and because the State has placed certain men in a board of health certain other men deem themselves justified in associating professionally with them. The State, also, through this same board, examines certain other men and gives them power to practice medicine. Now, if the State seal is curative in the one instance it is equally so in the other. If the legally qualified irregular may be consulted with in the State and national boards of health in reference to the public health, the legally qualified irregular may, also, be consulted with in reference to the health of the individual. There is an old and homely maxim, perhaps too homely for quotation here, about sauce which is *a propos.* Surely the sauce with which the regular member of the board of health is dressed, may be most appropriately used to garnish the private practitioner.

You will be able now, perhaps, to see that altered circumstances must modify the application of principles. Forty years ago the medical profession in this country stood alone. All laws regulating the practice of medicine had been repealed or were a dead letter. The profession stood upon its own merits, asking for and receiving no favors. If the great public did not choose to protect itself against ignorance and quackery the profession preferred to be calmly serene, rather than to appear to seek protection for itself. It could be as dignified and exclusive as it chose, and it chose to be both in an eminent degree. When the American Medical Association was organized, it, and the members of the profession at large, were free from all entangling alliances. Under such circumstances the section of the code regulating cousultations recommended itself to the judgment of the practitioner, and in its working there was no friction. Bnt, alas! it is so no longer. Wisely, or unwisely as individual judgment may pronounce it, alliances have been formed and their entangling complications are now embarrassing us.

Do you ask me for a solution of these difficulties? Admonished by the time which I have already occupied, I am reluctant to attempt one on this occasion. I shrink too from a task that has drawn down upon the State society of the Empire State the frowns of the American Medical Association.

But I may not shrink from giving you advice as to your conduct in reference thereto.

*First.* From an ethical standpoint be ever mindful of the fact that within the true Æsculapian fold you have unlimited freedom of medical creed and practice, and that a healthful and vigorous *esprit du corps*, which is the very soul of ethics, allows you to use the honorable title of Doctor of Medicine without qualifying designations of any kind whatever.

*Second.* Study thoroughly the code of the American Medical Association, and also the State laws relative to public health and the licensing power, and thus enable yourselves to form a clear and definite opinion as to "what's the matter."

*Third.* Promulgate that opinion on all suitable occasions, and be ever ready with your voice and vote to lend your aid in reconciliating conflicting conditions, by amendations and emendations both of the code and the State laws.

*Fourth.* Pending such changes, rendera llegiance to the code; so will you truly represent old Rush, your Alma Mater.

